# Transitional Cell Carcinoma of the Kidney Graft: An Extremely Uncommon Presentation of Tumor in Renal Transplant Recipients

**DOI:** 10.1155/2013/196528

**Published:** 2013-05-26

**Authors:** Vital Hevia, Victoria Gómez, Sara Álvarez, Víctor Díez Nicolás, Carmen Gómez del Cañizo, Andrea Orosa, Cristina Galeano Álvarez, F. J. Burgos Revilla

**Affiliations:** ^1^Department of Urology, Hospital Ramón y Cajal, Ctra. Colmenar km 9,100, 28034 Madrid, Spain; ^2^Department of Nephrology, Hospital Ramón y Cajal, Ctra. Colmenar km 9,100, 28034 Madrid, Spain

## Abstract

*Purpose*. Transitional cell carcinoma (TCC) affecting the graft after renal transplantation is a very infrequent way of presentation of this tumor. Our aim is to present our single institution experience with 2 cases, as well as to perform a review of the literature about this tumor after the transplant. *Materials and Methods*. TCC of the graft developed in 2 of 1365 patients from 1977 to 2010, both cases in women. Data were analyzed for incidence, clinical presentation, treatment, and outcomes. *Results*. Both cases occurred in 2 mid-age women and resulted to be high grade and locally advanced TCCs, representing an incidence of 0,14% (2/1365). Clinical presentation was urinary obstruction for the first case and incidental ultrasound finding for the second. Preoperative staging was made with CT, cytology, pyelography, ureterorenoscopy, and biopsy. Treatment performed was nephroureterectomy of the graft with bladder cuff and regional lymphadenectomy. Pathological examination showed in both cases a locally advanced and high grade urothelial carcinoma of the pelvis allograft. After 24 and 14 months of followup, both patients are disease free. *Conclusions*. TCC of the kidney graft is an infrequent tumor that has only been reported in a few cases in the literature. It usually appears at a lower age, more often locally advanced, and with poor differentiation. A multidisciplinary approach to treatment should be required in these cases.

## 1. Introduction

Nowadays, it is widely known that renal transplantation (RT) is the optimal treatment for end stage renal disease. However, after solid organ transplantation, there is a significant increased risk of developing a malignant neoplasm that varies based on cancer type [1]. This condition after RT is believed to depend on the length and type of immunosuppression or is associated with some viral infections. This risk is especially high for nonmelanoma skin cancer, hematological neoplasms, renal cell carcinoma, and thyroid cancer. Instead of this, prostate, testicular, or breast cancers have a lower risk [2]. Other tumors associated to viral infections and induced by that condition—such as hepatobiliary, cervical, or vulvovaginal neoplasms—have a higher incidence on transplant recipients.

Transitional cell carcinoma (TCC) of the urinary tract is one of those tumors that appear also more commonly on transplanted population than nontransplanted, varying its incidence according to the series from 0,07% [3] to 1,9% [4]. The possible cause for the increased risk of cancer in this population is due to immunosuppression, which decreases the host ability to defend against viral infections. In contrast, an important meta-analysis by Grulich et al. [5] compared the cancer risk in patients infected by HIV against patients who have received a solid organ transplant. TCC did not show an increased incidence in HIV population, so this finding could suggest that immunodeficiency would not be the leading and solitary cause for pathogenesis in this subgroup of neoplasms. Regarding the TCC, two main viruses had been related with an increased risk developing this neoplasm: BK virus (BKV) [6] and human papillomavirus (HPV) types 16 and 18 [7]. 

Most of these tumors are located in the bladder, but they can also appear at any location in the urinary tract: ureter, urethra, renal calyces, or pelvis. The risk increase for renal transplant recipients (RTRs) suffering a TCC has been estimated around 3 times higher than nontransplanted population [3]. Moreover, TCC in RTRs is regarded as being more aggressive, poorly differentiated, rapidly progressive, and more often fatal than in normal population. It is not surprising to find in these patients a higher incidence of locally advanced disease or metastatic presentation, as well as a significantly lower age at diagnosis [8, 9].

There are many reports and reviews in the literature about bladder TCC, assessing many aspects such as incidence, clinical presentation, treatment, or outcomes. Nevertheless, only a few cases of TCC affecting the renal allograft have been published. Our aim is to present our experience with 2 cases of TCC in the kidney allograft, as well as to perform a review of the existing literature about this extremely infrequent kind of presentation.

## 2. Patients and Methods

### 2.1. Case 1

**Figure 1:**
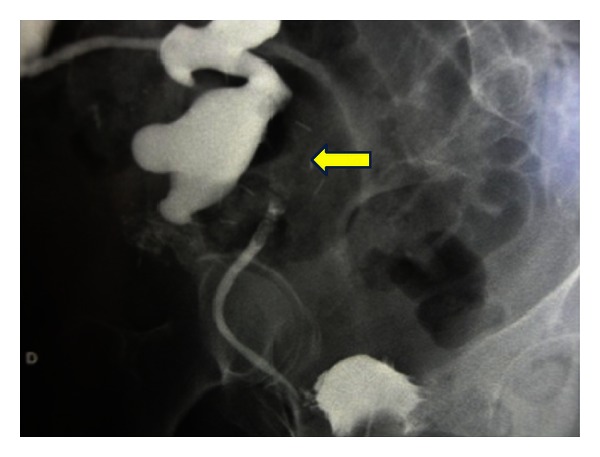
Antegrade pyelography showing a big filling defect in the kidney allograft secondary to a pelvis mass with moderate hydronephrosis.

A 61-year-old female patient developed chronic renal failure secondary to polycystic kidney disease and began hemodialysis in 1999. One year later she received a renal transplant from a deceased donor and underwent an immunosuppressive therapy based on corticoids, tacrolimus (FK506), and mycophenolate, with a mild chronic dysfunction at the moment of the diagnosis. After 12 years of transplantation, she came to the Emergency Department complaining of fever, urinary symptoms, and pain in the allograft region. An expansive process in allograft pelvis with secondary moderate hydronephrosis was detected on ultrasound, as well as higher serum creatinine levels. A percutaneous nephrostomy was placed, so creatinine returned to its baseline. Antegrade pyelography (Figure 1) revealed a big filling defect and a stop to the contrast at ureteropelvic junction, and the CT showed a big mass at renal pelvis that could infiltrate the adjacent fat. 

Selective urinary cytology and biopsy were positive for a high grade urothelial carcinoma. Inferior urinary tract was negative for tumor invasion. A nephroureterectomy of the renal allograft was performed. Histological analysis revealed a high grade urothelial carcinoma of the pelvis allograft with fat invasion and extension to proximal portion of the ureter, where it infiltrates the muscular layer. Surgical ureteral margin was free of tumor (stage pT3G4).

### 2.2. Case 2

**Figure 2:**
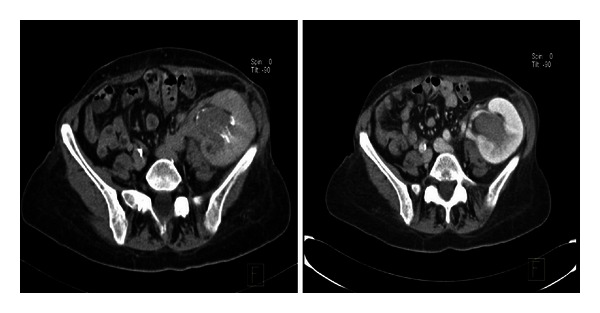
Pelvic CT demonstrating the presence of a big mass in the allograft, located in left iliac fossa, affecting renal pelvis and calyx.

A 59-year-old female patient developed chronic renal failure secondary to focal and segmental hyalinosis and began hemodialysis in 1987. In 1988 she underwent a renal transplantation but presented an acute rejection that required transplantectomy. A second RT was performed in 1991. After years she developed a chronic allograft dysfunction that made her return to hemodialysis in 2009, as well as undergoing another transplantectomy because of graft intolerance syndrome. Third RT was performed in 2010, using a renal allograft from a deceased male donor. Immunosuppressive therapy used was thymoglobulin, corticoids, tacrolimus (FK506), and mycophenolate. Immediate renal allograft function was achieved, and her baseline creatinine serum levels were around 1,2 mg/dL. After 14 months of third RT, in a routine review, ultrasound examinations revealed a heterogeneous renal mass located on inferior pole with moderate hydronephrosis associated. CT confirms the suspect (Figure 2) and shows a big mass in the kidney graft depending on the pelvis and probably infiltrating fat. 

**Figure 3:**
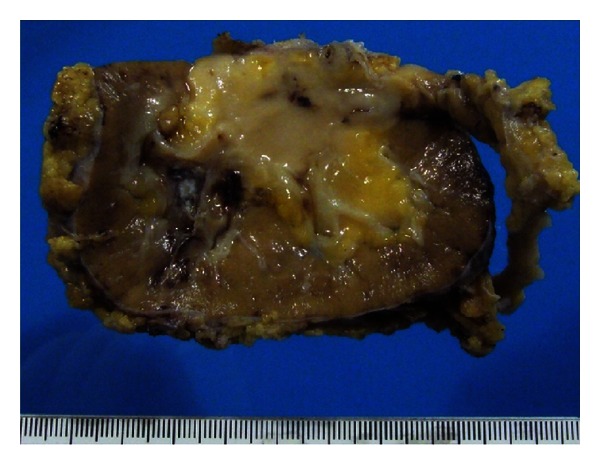
Macroscopic study after surgery that revealed a TCC of the renal pelvis with fat invasion and extension to proximal portion of the ureter, where it infiltrates the muscular layer. Surgical margin was free of tumor, and the stage was a pT3G4.

**Figure 4:**
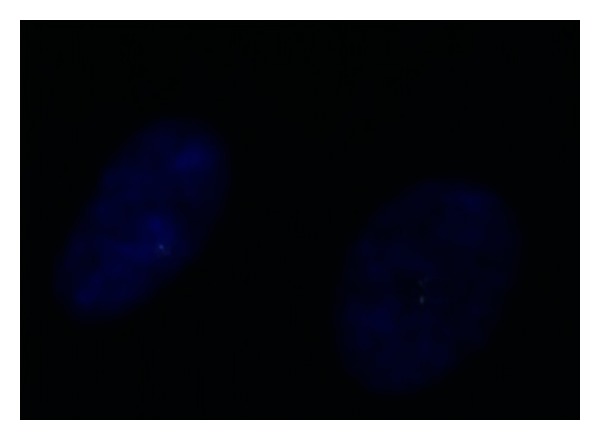
Cytogenetic analysis of the tumor's cells, demonstrating the presence of XY chromosomes.

Antegrade pyelography showed a big filling defect with amputation of medium and inferior calyxes. Selective urinary cytology was positive for a high grade urothelial carcinoma, and cystoscopy was negative for tumor invasion in the low urinary tract. Once again, a transplantectomy including ureter and bladder cuff was performed. Histological analysis revealed a high grade urothelial carcinoma of the pelvis allograft with fat invasion (Figure 3). Surgical ureteral margin was free of tumor (stage pT3(m)G3). Cytogenetic analysis of the tumor's cells (Figure 4) showed the presence of chromosomes XY, so it allowed us to establish the origin of the neoplasm in the donor cells, which was probably accelerated by high doses of immunosuppressants used.

Both patients had a successful postoperative period, returning to dialysis (first with hemodialysis and later with a peritoneal catheter) immediately. At the present moment, and after 24 and 14 months of followup with cystoscopy, urinary cytology, and CT, they are free of malignancy and no recurrence was diagnosed.

## 3. Discussion

The overall incidence of de novo malignancies after RT is about 4 to 5 times greater than in general population. Several malignancies do not show more risk, while others are significantly enhanced after transplantation [10]. Patients with an RT are at a higher risk of having a TCC compared to nontransplanted recipients, and it has been estimated around 3 times greater [3]. This fact, like many others related to epidemiology, varies substantially depending on the demographic area. Thus, there are series with an incidence of TCC in RTRs from 0,07% in University of California study [3] to 1,9% in Beijing group study [4]. This significant difference between the two regions is due in part to aristolochic acid, a substance in a Chinese herb which is known as an important risk factor of urothelial carcinoma [11] and was present as a risk factor in 16/27 (59,3%) of the RTRs diagnosed of TCC. 

Another important and widely known risk factor of TCC is Balkan endemic nephropathy (BEN), which is a primary renal disease that occurs in endemic regions of Croatia, Bulgaria, Bosnia, Romania, and Serbia. It produces end stage renal failure and is classically associated with 100 times greater incidence of TCC, especially in upper urinary tract [12, 13]. In fact, one of the few cases present in the literature of TCC in a renal allograft was in a Croatian 52-year-old male transplanted because of a CRF due to BEN. He first developed a low grade pTa TCC in left native kidney 3 years after RT (treated with left nephroureterectomy). Later, 6 years after RT, another low grade but pT2 TCC appeared in the allograft, limited to superior calyx. The uncommon of the case was the conservative treatment performed: a resection of the upper quarter of the allograft followed by a prophylactic right nephroureterectomy with bladder cuff resection. Cytopathological and genetic findings confirmed that the tumor arose from recipient urothelium and was not transferred by the donor. After 1-year followup, the patient maintained a normal renal function and was free of tumor recurrence [14].

Regarding clinical presentation of TCC in RTRs, it can occur in many ways. Painless gross hematuria is usually the most common symptom, as occurred in Li et al. [4] and Cox and Colli [9] studies. That is the main reason why some authors recommend a quick and thorough evaluation of gross hematuria [9, 15]. Other possibilities such as incidental finding, microhematuria, hydronephrosis, or irritative syndrome can be the first manifestation of a TCC. In the case of RTRs with the allograft affected by tumor, it is logical to think that hydronephrosis would be quite frequent, according to the high invasiveness of these tumors which can cause an obstruction of the urinary tract. Actually, the two cases presented hydronephrosis at the moment of diagnose, also with acute renal failure in case 1 due to obstructive uropathy.

High incidence of neoplasms and their aggressive course are associated with immunosuppressive therapy. Some immunosuppressant drugs such as cyclosporine (CsA) can promote cancer progression by a direct cellular effect [16]. Overimmunosuppression was also reported as one of factors related to the development of TCC [17], but it is still difficult to determine which dose of these drugs such as CsA or tacrolimus (FK506) is optimal and balanced between their side effects and their immunologic capacity. One last factor to consider is uremia. It is one of the preexisting factors that increase the risk of developing a TCC. Immunological dysfunction caused by uremia has been reported as an important factor involved in developing a TCC [2, 18].

Regarding to the location of TCC in RTRs population, bladder is the most common site in urinary tract where this tumor appears [4, 9]. It is followed by native upper urinary tract. Other locations reported in some articles are the urethra and the renal allograft, but much more infrequently. Actually, as commented previously, only a few cases of urothelial cancer in the allograft have been reported in the literature [6, 9, 14, 19, 20].

Another important fact is that maybe due to immunosuppression, the tumor stage at diagnosis is frequently more locally advanced, poorly differentiated, and with a lower age at the time of presentation [10].This is the reason why some groups recommend an exhaustive followup and evaluation of these patients in order to prevent aggressive clinical presentations of TCC, especially if they complain of gross hematuria [9].

The way of treatment of TCC in RTRs is not so different from the way in nontransplanted population. In the low urinary tract, it goes from bladder transurethral resection (TURB) for nonmuscle-invasive bladder cancer (NMIBC) to radical cystectomy (RC) and Urinary diversion for muscle-invasive bladder cancer (MIBC). TCCs of native upper urinary tract are classically treated with radical nephroureterectomy (RNU); sometimes it is performed as a prophylactic treatment in high risk cases. In those cases that the TCC appears affecting the renal allograft, the standard treatment should be the radical transplantectomy including the ureter and a bladder cuff, considering as an option for selected cases with low grade tumors the percutaneous resection. Mokos et al. [14] report a case treated with partial nephrectomy including the upper quarter of the allograft in a low grade TCC with *muscularis propria * invasion of the superior calyx, being free of recurrence after 1 year followup. It could maybe represent a less safety treatment from an oncological point of view, but the approach should consider also every medical aspect such as the return to dialysis. Because of particularities of TCC in RTRs already discussed, such as higher grade, multicentricity, more locally advanced and more metastatic presentation, radical nephroureterectomy of the allograft including ureter and bladder cuff would be the standard of treatment, even knowing that it would make the patient return to dialysis.

## 4. Conclusion

Transitional cell carcinoma, such as other malignancies, appears more commonly in renal transplant recipients than in nontransplanted population. Gross hematuria is the more frequent symptom of the TCC, but not the only one. The renal allograft is a very uncommon location of TCC; bladder is the most often place in the urinary tract. They usually appear at a lower age and are often more locally advanced, poorly differentiated, and with metastatic disease. Proper treatments should be tailored to each case according to a multidisciplinary approach, including urologists, nephrologists, and oncologists.
